# Data on the early oxidation of SiO_2_-coated pure Ti and bulk Ti_5_Si_3_ at 800 °C

**DOI:** 10.1016/j.dib.2018.08.173

**Published:** 2018-09-01

**Authors:** Kathleen Chou, Peng-Wei Chu, Emmanuelle A. Marquis

**Affiliations:** Department of Materials Science and Engineering, University of Michigan, Ann Arbor, MI 48109, USA

## Abstract

Oxidation of pure Ti sputtered with a 250 nm layer of amorphous SiO_2_ and bulk Ti_5_Si_3_ was conducted at 800 °C for 2 or 32 h in a 1 standard cubic centimeter per minute (SCCM) O_2_/4 SCCM Ar environment (approximately pO_2_ = 0.2 atm/20.3 kPa). Specimens were characterized using transmission electron microscopy, scanning transmission electron microscopy, and energy dispersive spectroscopy. The data in this article accompanies research article “Early oxidation behavior of Si-coated titanium” [1], which contains further discussion. The data for this article is hosted at the Materials Commons data repository and is available for download at https://materialscommons.org/mcapp/#/data/dataset/b8bc8038-a735-4cb9-9a9e-a0fb912b248c.

**Specifications Table**

**Table:** 

Subject area	Materials Science
More specific subject area	Titanium Oxidation
Type of data	Figures
How data was acquired	Scanning electron microscopy (Thermo Fisher Scientific FEI Helios 650 Nanolab), Transmission electron microscopy (JEOL 2010F), Scanning transmission electron microscopy (Hitachi HD-2300A) with energy dispersive spectroscopy (Oxford Instruments)
Data format	Image files of analyzed data
Experimental factors	Sputtering of the 250 nm amorphous SiO_2_ layer on pure Ti was performed using a Kurt J. Lesker Co. five source confocal, magnetron sputtering system. Bulk Ti_5_Si_3_ was synthesized using arc melting.
Experimental features	Oxidation exposures for pure Ti with a 250 nm layer of amorphous SiO_2_ and bulk Ti_5_Si_3_ for 2 or 32 h at 800 °C in a 1 standard cubic centimeter per minute (SCCM) O_2_/4 SCCM Ar environment (approximately pO_2_ = 0.2 atm/20.3 kPa).
Data source location	Ann Arbor, MI, USA (42.2995°N, 83.7076°W)
Data accessibility	The data for this article is hosted at the Materials Commons data repository and is available for download at https://materialscommons.org/mcapp/#/data/dataset/b8bc8038-a735-4cb9-9a9e-a0fb912b248c

**Value of the data**

• This dataset demonstrates the early oxidation behavior of pure Ti sputtered with a SiO_2_ film to supplement the interpretation of early oxidation behavior of pure Ti sputtered with a Si film.
• This dataset demonstrates differences in the early oxidation behavior of bulk Ti_5_Si_3_ compared to early oxidation behavior of pure Ti sputtered with a Si film.
• Data serves as a reference in the development of further experiments for oxidation studies of Ti with silicon-containing coatings.

## 1 Data

The data in this article contains bright field transmission electron microscopy (TEM) images, scanning transmission electron microscopy (STEM) images, selected area diffraction patterns, and elemental maps from energy dispersive spectroscopy (EDS). This data was collected from oxidized specimens of pure Ti sputtered with a 250 nm layer of amorphous SiO_2_ and oxidized specimens of bulk Ti_5_Si_3_.

## 2 Experimental design, materials and methods

### 2.1 Oxidation exposure procedure

The oxidation of pure Ti specimens sputtered with a 250 nm layer of amorphous SiO_2_ was conducted for 2 or 32 h exposures at 800 °C in a 1 standard cubic centimeter per minute (SCCM) O_2_/4 SCCM Ar environment (approximately pO_2_ = 0.2 atm/20.3 kPa) using a Thermo Scientific Lindberg Blue M tube furnace. The exposures were such that specimens were inserted in the hot zone of the furnace after it was heated to 800 °C in a flowing Ar (40 SCCM) gas environment. After insertion and temperature equilibration back to 800 °C (approximately 15 min), the aforementioned oxidizing environment was introduced. Following the oxidation exposure, oxygen gas flow was stopped. Specimens were removed from the hot zone and cooled to room temperature in flowing Ar (40 SCCM). Sputtering of the amorphous SiO_2_ layer was performed using a Kurt J. Lesker Co. five source confocal, magnetron sputtering system. Additionally, the oxidation of bulk Ti_5_Si_3_ was conducted for a 32 h exposure at 800 °C in a 1 SCCM O_2_/4 SCCM Ar environment using the same procedure. The bulk Ti_5_Si_3_ sample was synthesized through arc melting of bulk Ti and Si pieces. The resulting bulk Ti_5_Si_3_ sample prior to oxidation exposure had an as-cast microstructure consisting of Ti_5_Si_3_ and α Ti.

### 2.2 Characterization methods

Cross-sectional transmission electron microscopy (TEM) foils of the coating and oxide were prepared using a Thermo Fisher Scientific FEI Helios 650 Nanolab scanning electron microscope (SEM) equipped with a focused ion beam (FIB). TEM bright field images and selected area electron diffraction (SAED) patterns were obtained using a JEOL 2010F microscope operated at 200 kV. Bright field TEM figures are composites of multiple individual specimen images. Scanning transmission electron microscope (STEM) images and energy dispersive spectroscopy (EDS) maps were collected using a Hitachi HD-2300A microscope at 200 kV equipped with an Oxford Instruments EDS detector. EDS maps were collected using an acquisition time of 1800 s. Data visualization and analysis of EDS maps were performed using Oxford Instruments INCA software.

### 2.3 Results

**Fig. 1:**
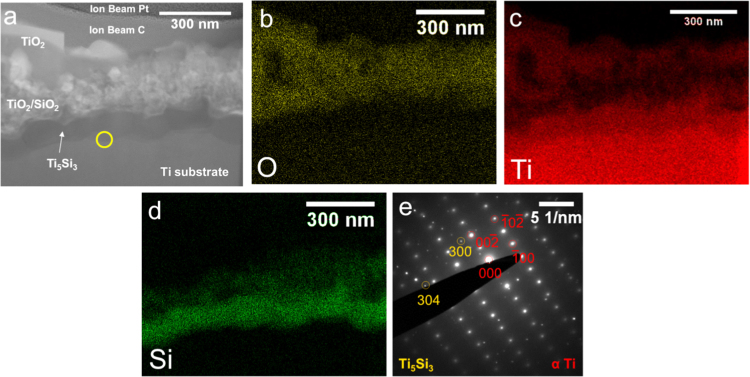
(a) STEM diffraction contrast image and associated EDS maps for O, Ti, and Si (b-d) of the coating and oxide cross section for SiO_2_-coated Ti oxidized at 800 °C for 2 h. (e) Selected area diffraction pattern at location of yellow circle in (a), indexed as Ti_5_Si_3_ and α Ti.

**Fig. 2:**
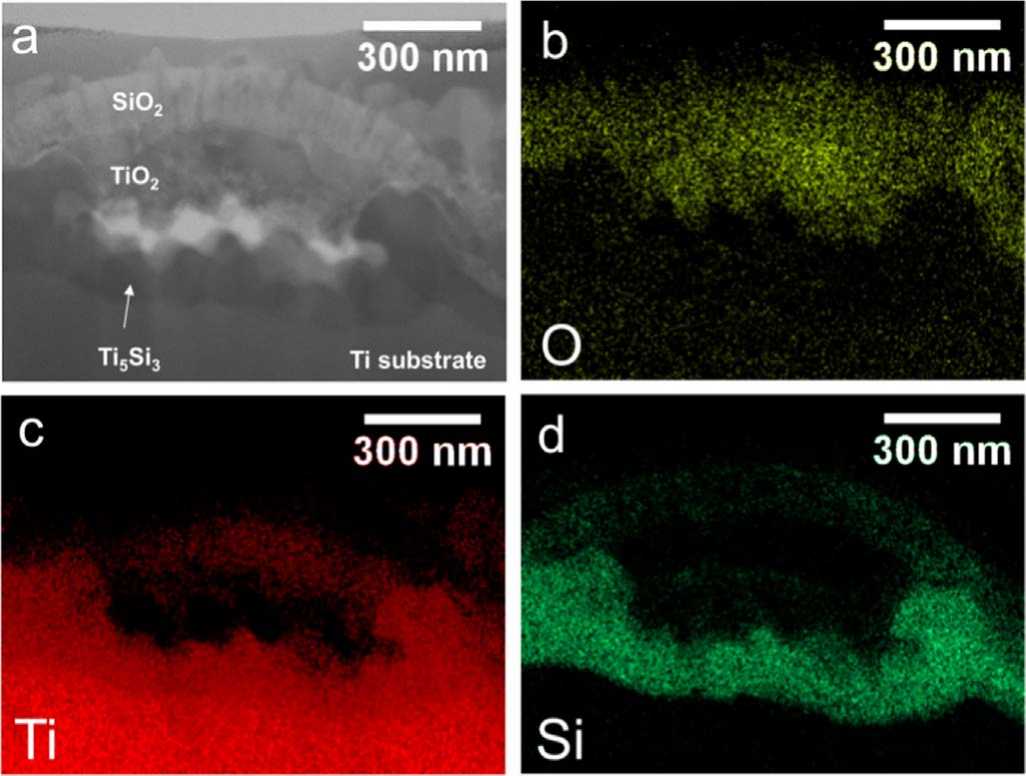
(a) STEM diffraction contrast image and associated EDS maps for O, Ti, and Si (b-d) at a delamination in the coating and oxide layers for SiO_2_-coated Ti oxidized at 800 °C for 2 h.

The coating and oxide structure for SiO_2_-coated Ti oxidized for 2 h (Fig. 1, Fig. 2) showed similar layers to a Si-coated Ti specimen also oxidized at 800 °C for 2 h [1]. A compact and polycrystalline layer, identified as Ti_5_Si_3_ through EDS mapping and selected area electron diffraction patterns, was observed adjacent to the Ti metal. This was covered by a two-layer scale that contained an inner equiaxed, nanocrystalline layer containing TiO_2_ and SiO_2_ and outermost layer of TiO_2_ oxide crystals, identified through EDS mapping. Delamination of the SiO_2_ layer was observed in some areas at the SiO_2_/Ti_5_Si_3_ interface (Fig. 2a); however, in contrast with Si-coated Ti specimens in the accompanied study [1], no outward bowing of the SiO_2_ layer was observed.

**Fig. 3:**
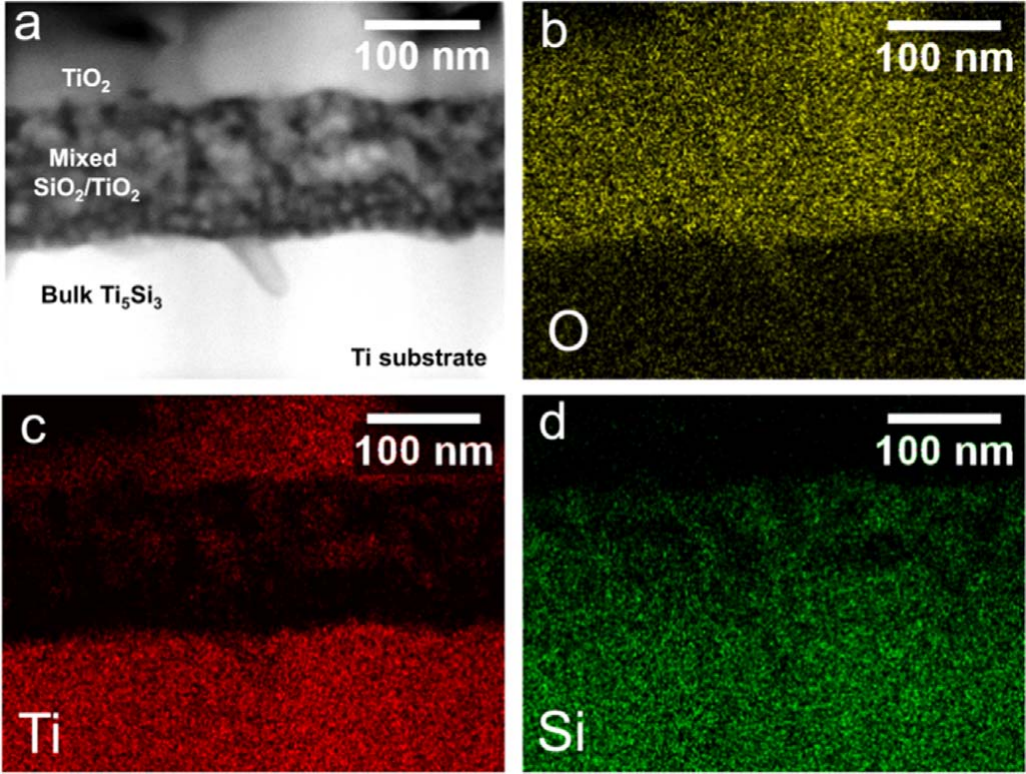
(a) STEM Z-contrast image and associated EDS maps for O, Ti, and Si (b-d) for bulk Ti_5_Si_3_ oxidized at 800 °C for 32 h.

STEM imaging and EDS mapping of oxidized bulk Ti_5_Si_3_ for 32 h (Fig. 3) showed a two-layer scale: the outermost scale was Ti-rich and corresponded to TiO_2_ and the internal scale showed mixed Ti-rich and Si-rich regions, corresponding to TiO_2_ and SiO_2_. In contrast with oxidation of Si-coated Ti [1], no regular patterning of alternating SiO_2_ and TiO_2_ layers was observed for oxidized Ti_5_Si_3_.

**Fig. 4:**
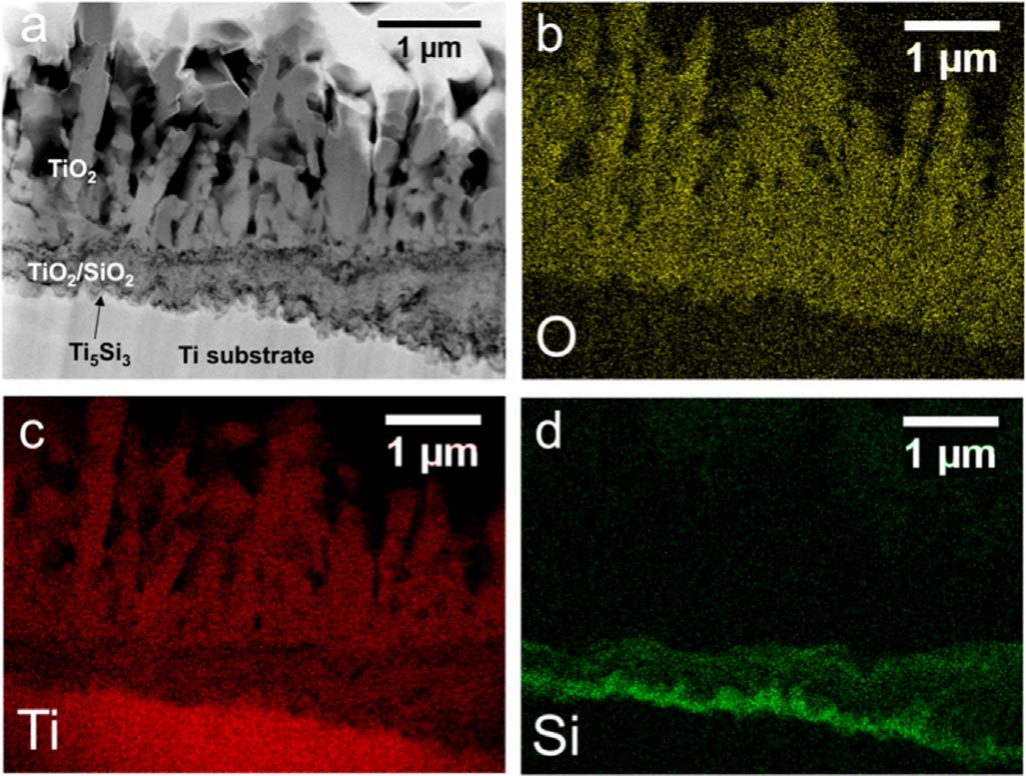
(a) STEM Z-contrast image and associated EDS maps for O, Ti, and Si (b-d) of the coating and oxide cross section for SiO_2_-coated Ti oxidized at 800 °C for 32 h.

**Fig. 5:**
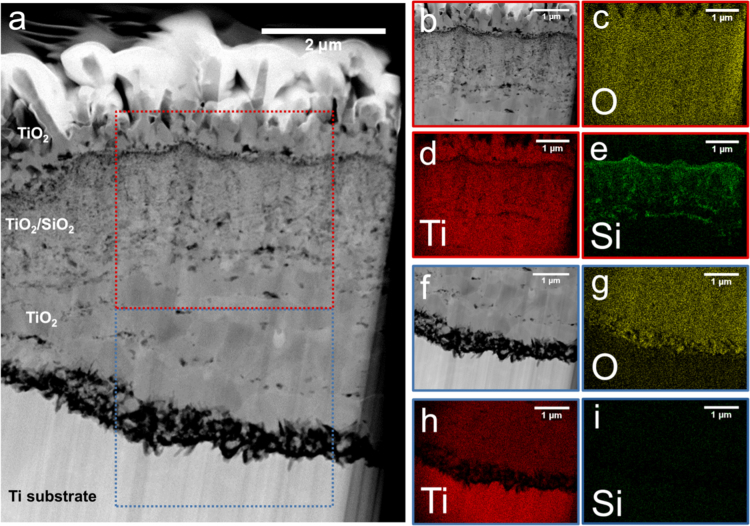
(a) STEM Z-contrast image for SiO_2_-coated Ti oxidized at 800 °C for 32 h. (b) STEM Z-contrast image for subset outlined by red box and (c-e) associated EDS maps for O, Ti, and Si showing Si distributed within the internal oxide. (f) STEM Z-contrast image for subset outlined by blue box and (g-i) associated EDS maps for O, Ti, and Si, in which Si is not detected at the oxide/metal interface.

Oxidation of the SiO_2_-coated Ti specimens for 32 h revealed locations with distinctly different scale morphologies and internal oxide thicknesses (Fig. 4, Fig. 5). An outward growing wedge-like external scale comprised of rutile TiO_2_ was observed at all locations in the cross section. The presence of the Ti_5_Si_3_ layer between the internal oxide and Ti substrate, identified through EDS mapping, corresponded to regions with a thin internal nanocrystalline oxide scale (Fig. 4), which was similar to Si-coated Ti oxidized specimens [1]. In regions where the Ti_5_Si_3_ layer was no longer observed, the internal oxide was much thicker (Fig. 5). Si was detected through EDS mapping at the top of the internal oxide, but the underlying oxide contain very little Si signal and showed a much larger grain size. The lack of Si in the large grained portion of the internal oxide indicates that the Ti_5_Si_3_ layer had been fully oxidized and was no longer contributing to oxidation behavior of the Ti substrate.
